# Autophagy preserves hematopoietic stem cells by restraining MTORC1-mediated cellular anabolism

**DOI:** 10.1080/15548627.2023.2247310

**Published:** 2023-08-23

**Authors:** Mariana Borsa, Sandrine Obba, Felix C. Richter, Hanlin Zhang, Thomas Riffelmacher, Joana Carrelha, Ghada Alsaleh, Sten Eirik W. Jacobsen, Anna Katharina Simon

**Affiliations:** a Kennedy Institute of Rheumatology NDORMS, University of Oxford, Oxford, UK; b MRC Molecular Haematology Unit, MRC WIMM, Radcliffe Department of Medicine, University of Oxford, Oxford, UK; c H7 Department of Medicine, Karolinska Institute, Stockholm, Sweden; d Max Delbrück Center for Molecular Medicine, Berlin, Germany

**Keywords:** Autophagy, amino acids, hematopoietic stem cells, MTOR, rapamycin, translation

## Abstract

Adult stem cells are long-lived and quiescent with unique metabolic requirements. Macroautophagy/autophagy is a fundamental survival mechanism that allows cells to adapt to metabolic changes by degrading and recycling intracellular components. Here we address why autophagy depletion leads to a drastic loss of the stem cell compartment. Using inducible deletion of autophagy specifically in adult hematopoietic stem cells (HSCs) and in mice chimeric for autophagy-deficient and normal HSCs, we demonstrate that the stem cell loss is cell-intrinsic. Mechanistically, autophagy-deficient HSCs showed higher expression of several amino acid transporters (AAT) when compared to autophagy-competent cells, resulting in increased amino acid (AA) uptake. This was followed by sustained MTOR (mechanistic target of rapamycin) activation, with enlarged cell size, glucose uptake and translation, which is detrimental to the quiescent HSCs. MTOR inhibition by rapamycin treatment *in vivo* was able to rescue autophagy-deficient HSC loss and bone marrow failure and resulted in better reconstitution after transplantation. Our results suggest that targeting MTOR may improve aged stem cell function, promote reprogramming and stem cell transplantation.

**List of abbreviations:** 5FU: fluoracil; AA: amino acids; AKT/PKB: thymoma viral proto-oncogene 1; ATF4: activating transcription factor 4; BafA: bafilomycin A_1_; BM: bone marrow; EIF2: eukaryotic initiation factor 2; EIF4EBP1/4EBP1: eukaryotic translation initiation factor 4E binding protein 1; KIT/CD117/c-Kit: KIT proto-oncogene receptor tyrosine kinase; HSCs: hematopoietic stem cells; HSPCs: hematopoietic stem and progenitor cells; Kyn: kynurenine; LSK: lineage^−^ (Lin^−^), LY6A/Sca-1^+^, KIT/c-Kit/CD117^+^; LY6A/Sca-1: lymphocyte antigen 6 family member A; MTOR: mechanistic target of rapamycin kinase; MTORC1: MTOR complex 1; MTORC2: MTOR complex 2; OPP: O-propargyl-puromycin; PI3K: phosphoinositide 3-kinase; poly(I:C): polyinosinic:polycytidylic acid; RPS6/S6: ribosomal protein S6; tam: tamoxifen; TCA: tricarboxylic acid; TFEB: transcription factor EB; PTPRC/CD45: Protein Tyrosine Phosphatase Receptor Type C, CD45 antigen.

## Introduction

A fine balance of self-renewal and differentiation fate choices allows hematopoietic stem cells (HSCs) to maintain themselves and produce lineage restricted progenitors to fulfil the demands of the blood system. Evidence is accumulating that healthy HSCs only survive and function properly if they maintain a balanced metabolic state. This is achieved by restrained activation of the MTOR (mechanistic target of rapamycin kinase), which orchestrates catabolism (break-down) and anabolism (growth and building) [1]. Regulation of downstream targets by MTOR signaling is mediated through the action of two complexes, MTOR complex 1 (MTORC1) and MTOR complex 2 (MTORC2). MTORC1 activity promotes cell growth, glycolysis, increased protein and lipid biosynthesis, but restricts macroautophagy/autophagy and lysosome biogenesis [2]. Protein synthesis is regulated through the phosphorylation of RPS6/S6 (ribosomal protein S6) and the EIF4EBP1/4EBP1 (eukaryotic translation initiation factor 4E binding protein 1), and thereby controls the initiation of protein translation [3]. Active MTORC1 inhibits autophagy by phosphorylation-dependent inhibition of ATG13 and ULK1 and by inhibiting the activity of TFEB (transcription factor EB) family members [3]. Restrained PI3K (phosphoinositide 3-kinase)-AKT/PKB (thymoma viral proto-oncogene 1)-MTOR signaling promotes autophagy and is essential for maintaining functional and long-lived HSCs endowed with capacity to both differentiate and self-renew [2]. In contrast, elevated MTORC1 activity is detrimental to stemness and is linked to HSC premature aging and depletion [1,3].

Adult HSCs are mostly quiescent with low energy requirements [4]. They rely on glycolysis [5,6] and fatty acid oxidation [7], as opposed to downstream progenitors that use mitochondrial oxidative phosphorylation [8]. Lower protein synthesis rates in adult HSCs have been linked to their state of quiescence when compared to rapidly dividing progenitors [9,10]. Moreover, increased protein synthesis impairs stem cell function [11].

Interestingly, several amino acids (AA) have emerged as key regulators of HSC function. AA are found in much higher concentrations in the bone marrow (BM) than in peripheral blood, which itself suggests they might play an important role in hematopoiesis [12,13]. Valine has been shown to be critical for mouse and human HSC proliferation and for retention in the BM [14]. Glutamine uptake and metabolism allow hematopoietic stem and progenitor cell (HSPC) differentiation into erythroid lineage and for *de novo* nucleotide synthesis [15]. The availability of leucine, an essential AAs, is believed to regulate nutrient sensing pathways, such as the MTOR pathway, in HSCs [2]. Essential AAs are either recycled or acquired by means of dietary intake and transported into cells through the plasma membrane-spanning transporters [16]. Free intracellular AAs not only serve as a source of metabolites for protein synthesis and energy, but also directly contribute to the tight regulation of two pathways, the MTORC1 and EIF2 (eukaryotic initiation factor 2) cascades that integrate anabolic and catabolic signals. Moreover, nutrient availability regulates the integrated stress response (ISR) pathways, which are believed to promote the survival of healthy HSPCs [17].

Autophagy is one of the main catabolic pathways in the cell that degrades cellular constituents specifically to be re-used as building blocks. Together with synthesis, import and proteasomal degradation, autophagy contributes to intracellular AA availability. Considerable evidence has accumulated over the last years supporting a key role for autophagy in the maintenance and function of HSCs [18,19]. Conditional deletion of *Atg7* in all hematopoietic lineages results in significantly reduced HSC numbers, accompanied by their excessive differentiation into lineage^−^ (Lin^−^), LY6A/Sca-1 (lymphocyte antigen 6 family member A)^+^, KIT/CD117/c-Kit (KIT proto-oncogene receptor tyrosine kinase)^+^ (LSK) progenitor populations, enlarging that compartment [18]. Additionally, LSK progenitor cells show a significant accumulation of mitochondria, reactive oxygen species (ROS), DNA damage and hallmarks of apoptosis [18]. Similarly, inducible pan-hematopoietic *Atg12* deletion results in increased mitochondrial content and changes in HSC metabolism [19,20]. Both *atg7-* and *atg12-* deficient HSCs exhibit impaired self-renewal and regenerative potential. However, the pan-hematopoietic deletion of *Atg7* and *Atg12* using *Vav*^Cre^ and *Mx1*^*Cre*^-driven deletion, respectively, has important limitations. Pan-hematopoietic autophagy deletion by *Vav*^*Cre*^ results in peripheral cytopenia since maintenance of mature blood cell lineages is also regulated by autophagy, which restricts the interpretation of the direct impact of autophagy in HSCs [18]. *Mx1*^*Cre*^ additionally targets other non-hematopoietic cell types, and requires treatment with polyinosinic:polycytidylic acid (poly(I:C)), which can additionally affect the HSC phenotype [19].

Here, we show that depleting autophagy in non-cytopenic conditions led to a cell-intrinsic decline in HSC numbers. In contrast, the expansion of the early LSK multipotent progenitor compartment was found to be mediated cell-extrinsically. We then investigated which molecular pathways in autophagy-deficient HSCs lead to their depletion. We observed that autophagy-deficient HSCs showed higher expression of several AA transporters when compared to autophagy-competent cells, resulting in increased AA uptake and culminating in enhanced MTOR activation. This led to detrimental functional consequences within HSCs, mediating increased cell size, glucose uptake, and protein synthesis. Importantly, the numbers, reconstitution potential and metabolic health of HSCs could be restored upon rapamycin treatment *in vivo*. Together, this suggests that increased MTOR activation is a maladaptation of autophagy-deficient HSCs resulting from excessive influx of AAs.

## Results

### HSC homeostasis is dependent on cell intrinsic autophagy

**Figure 1.**
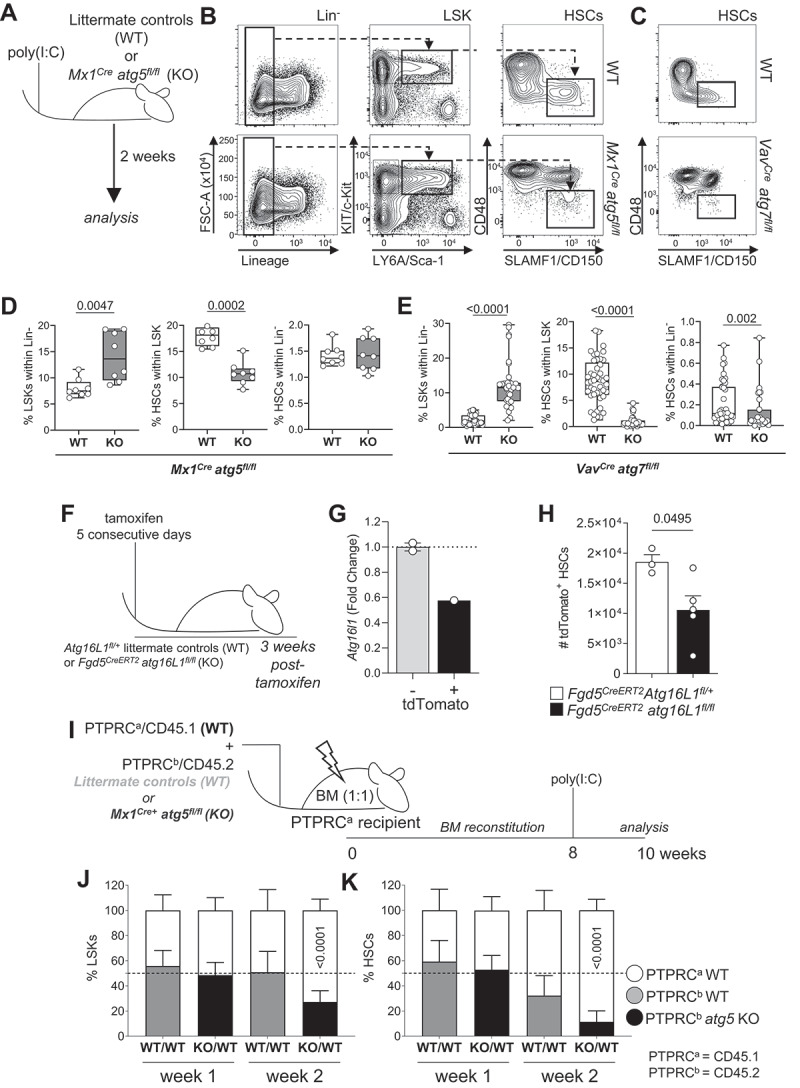
HSC homeostasis is dependent on cell intrinsic autophagy. (A) Experimental setup for the inducible deletion of *Atg5* in hematopoietic cells (*Mx1*^*Cre*^ *atg5*^*fl/fl*^). (B) Gating strategy: representative dot plots of littermate controls (*WT*) and *Mx1*^*Cre*^ *atg5*^*fl/fl*^ SLAMF1/CD150^+^ CD48^−^ (HSCs) gated on Lin^−^ LY6A/Sca-1^+^ KIT/cKit^+^ (LSK) BM cells 2 weeks after poly(I:C) treatment. (C) Representative dot plots of *WT* and *Vav*^*Cre*^ *atg7*^*fl/fl*^. (D) LSK frequency within the Lin^−^ population (left panel), HSC frequency within LSK population (middle panel) and HSC frequency within Lin^−^ population (right panel) in *Mx1*^*Cre*^ *atg5*^*fl/fl*^ (*n* = 8 mice/group). Data are represented as mean ± SEM with Mann-Whitney test. (E) LSK frequency within the Lin^−^ population (left panel), HSC frequency within LSK population (middle panel) and HSC frequency within Lin^−^ population (right panel) in *Vav*^*Cre*^ *atg7*^*fl/fl*^ (*n* = 45 mice/group). Data are represented as mean ± SEM with Mann-Whitney test. (F) Experimental setup of the tamoxifen-inducible deletion of *Atg16l1* and Tomato reporter (*Ai14*) expression by *Fdg5*^*Cre*^. (G) *Atg16l1* transcript levels in Tomato^+^ LSKs. (H) Total number of Tomato^+^ HSCs (left and right hip and leg bones) three weeks post-tamoxifen treatment. Data are represented as mean ± SEM with two-tailed unpaired Student’s *t* test. Representative data from 1 out of 2 experiments (n_WT_ = 3, n_KO_ = 5). (I) Experimental setup for the generation of mixed bone marrow chimeras inducible for deletion of *Atg5* (*Mx1*^*Cre*^ *atg5*^*fl/f*l^). Lethally irradiated PTPRC^a^/CD45.1 hosts reconstituted with a 1:1 mix of BM of *Mx1*^*Cre*^ *atg5*^*fl/fl*^ or *WT* BM (PTPRC^b^/CD45.2) and PTPRC^a^/CD45.1 *WT* BM. After 8 weeks, *Atg5* deletion was induced by poly(I:C) and BM was analyzed at indicated times points after poly(I:C) administration. (J) Frequencies of PTPRC^a^/CD45.1 cells and PTPRC^b^/CD45.2 LSKs within Lin^−^ cells from mice reconstituted with PTPRC^a^/CD45.1 *WT*:PTPRC^b^/CD45.2 *WT* (white/gray) or with PTPRC^a^/CD45.1 *WT*:PTPRC^b^/CD45.2 *Mx1*^*Cre*^ *atg5*^*fl/fl*^ (white/black) BM. (K) Frequencies of HSCs within LSK in BM chimera. (J, K) data represented as mean ± SEM. *n* = 3–4 mice. Two-way ANOVA with post hoc Sidak’s test.

We first confirmed the relevance of autophagy for HSC homeostasis. Autophagy was deleted by the *Mx1*^*Cre*^-driven *Atg5* excision following poly(I:C) injection (Figure 1A) [19] or *Atg7* was deleted using the pan-hematopoietic *Vav*^*Cre*^ model [18]. HSCs were defined as LSK, as well as CD48^−^ SLAMF1/CD150^+^ (Figure 1B,C). In both models, we observed an increase in the LSK population containing primarily multipotent progenitors, whereas HSCs were dramatically reduced (Figure 1D,E), in agreement with previous studies [18,19]. In these models, genetic deletion is driven by Cre expression from strong promoters affecting all hematopoietic cells (*Vav*^*Cre*^ or *Mx1*^*Cre*^). As loss of autophagy also results in extensive depletion of most mature blood cell lineages such as erythrocytes [21], platelets [22], and T cells [23], leading to cytopenia in the periphery, HSCs and their progenitors will be activated to replenish these lineages. Hence, it remained unclear to what degree these and previously reported reductions in HSC numbers and changes in functional parameters such as metabolism, mitochondrial health, self-renewal, differentiation, and bone marrow failure, could be a consequence of peripheral cytopenia rather than due to a direct intrinsic effect of autophagy-deficiency in HSCs. Moreover, deleting autophagy in all IFNα-responsive cells using *Mx1*^*Cre*^ [24] also affects other cells such as bone marrow stromal cells [25]. To avoid cytopenia and to be able to differentiate cell-extrinsic from HSC-intrinsic roles of autophagy, we therefore evaluated the impact of autophagy loss through inducible autophagy deletion in mixed BM chimera settings, or where deletion is restricted to HSCs.

Firstly, we decided to take advantage of the *Fgd5*^*CreERT2*^ model [26], which drives tamoxifen-inducible *Atg16l1* deletion specifically in HSCs (*Fdg5*^*CreERT2*^ *atg16l1*^*fl/fl*^ Rosa26-stop-tdTomato, here after named *Fdg5*^*CreERT2*^ *atg16l1*^*fl/fl*^) without leading to cytopenia (Figure 1F, Fig. S1A). tdTomato/Tomato expression was used as an indicator of *Atg16l1* deletion in this model. Tomato^+^ LSK cells from *Fdg5*^*CreERT2*^ *atg16l1*^*fl/fl*^ mice showed a 40% reduction in *Atg16l1* transcripts in comparison to Tomato^−^ cells isolated from the same animals (Figure 1G). We also confirmed that this reflected in lower autophagy flux (Fig. S1B). This led to a 40–50% decrease in the absolute number of Tomato^+^ HSCs in *Fgd5*^*CreERT2*^ *atg16l1*^*fl/fl*^ mice when compared to *Fgd5*^*CreERT2*^*Atg16L1*^*fl/+*^ littermates (Figure 1H), suggesting that autophagy plays a cell-intrinsic role in HSCs maintenance.

We next mixed PTPRC^b^/CD45.2 BM cells from *Mx1*^*Cre*^ *atg5*^*fl/fl*^ or *Mx1*^*Cre*^ *Atg5*^*fl/+*^ littermate controls with PTPRC^a^/CD45.1 BM cells from *WT* mice (1:1) to reconstitute the hematopoietic system of an irradiated host mouse (Figure 1I). Efficient *Atg5* deletion was confirmed by qPCR (Fig. S1C). Frequency of *atg5*-deficient LSK cells were reduced in chimeric mice (Figure 1J), demonstrating that the LSK expansion observed in the non-chimeric *Vav*^*Cre*^ and *Mx1*^*Cre*^ models (Figure 1D,E) is mediated by HSC-extrinsic mechanisms. Importantly, *atg5*-deficient HSCs were reduced by week 2 following inducible deletion of *Atg5* in the chimeric setting (Figure 1K), reinforcing the point that HSC homeostasis relies on intact autophagy. Taken together, these findings indicate that HSCs loss driven by autophagy deletion is a cell-autonomous mechanism.

### Autophagy deletion leads to increased expression of AA transporters and AA uptake

**Figure 2.**
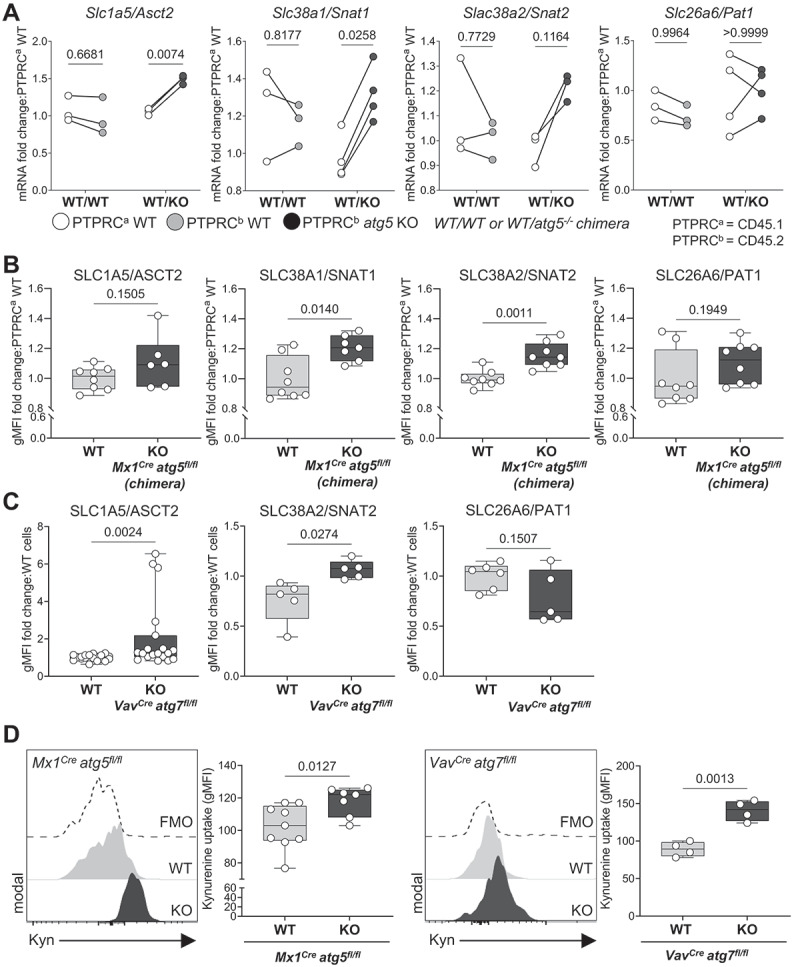
Autophagy deletion leads to increased expression of AA transporters and AA uptake in HSCs. (A) Real-time qPCR analysis of gene expression of amino acid transporters (AATs) in sorted LSKs from *Mx1*^*Cre*^ *atg5*^*fl/fl*^ chimera (as in Figure 1I). Data are relative to *Actb* (*n* = 3–4/group). Two-way ANOVA with post hoc Sidak’s test. (B) The geometric mean of fluorescence (gMFI) of surface AAT in *Mx1*^*Cre*^ LSK cells from chimeric mice was normalized and expressed in fold change to corresponding *WT* cells (*n* = 6–8 mice/group). Data are represented as mean ± SEM with Mann-Whitney test. (C) gMFI of SLC1A5/ASCT2, SLC38A2/SNAT2 and SLC26A6/PAT1 in HSCs from *Vav*^*Cre*^ *atg7*^*fl/fl*^ mice is normalized and expressed in fold change to littermate controls (*WT*). n_WT_ = 17–20 and n_KO_ = 8–19 per group). Data are represented as mean ± SEM with Mann-Whitney test. (D) Representative histograms and gMFI of Kyn uptake in HSCs from *Mx1*^*Cre*^ *atg5*^*fl/fl*^ mice (left, n_wt_ = 9, n_KO_ = 7) and *Vav*^*Cre*^ *atg7*^*fl/fl*^ mice (right, *n* = 4). Data are represented as mean ± SEM with Mann-Whitney test. FMO, fluorescence minus one.

Previous studies found an increased mitochondrial mass in autophagy-deficient HSCs, concluding that mitochondria are no longer degraded by autophagy, and suggesting that this contributes to HSC loss [18,19]. However, HSCs rely mostly on glycolysis with little contribution from mitochondrial respiration to meet their energetic demands [5,7,8]. Given that AA are key for HSC function and survival [12], we investigated the consequences on AA supply in HSCs when autophagy is deficient. We observed that both gene and protein surface expression of the major AA transporters of glutamine, leucine, and arginine (SLC1A5/ASCT2, SLC38A1/SNAT1, SLC38A2/SNAT2) were upregulated in *atg5* KO HSCs isolated from BM chimeras (Figure 2A, B). This result was confirmed in HSCs in which *Atg7* (Figure 2C) or *Atg16l1* were deleted (Fig. S1D and Fig. S2A-B). For the latter, in contrast to our observations in *atg5* KO HSCs from BM chimeras and *atg7* KO cells, we found SLC26A6/PAT1 to be upregulated in HSCs (Fig. S2B). Functionally, upregulation of surface expression of AA transporters resulted in an increase in AA uptake in autophagy-deficient HSCs (*Atg5*^*Mx1-Cre*^ or *Atg7*^*Vav-Cre*^ deletion) as measured by the flow cytometry-based kynurenine (Kyn) assay [27] (Figure 2D). Overall, our data suggest that autophagy loss results in upregulation of AA transporters and increased influx of AA.

### Increased AA uptake results in MTOR activation, cell growth, upregulation of glycolysis and protein translation

**Figure 3.**
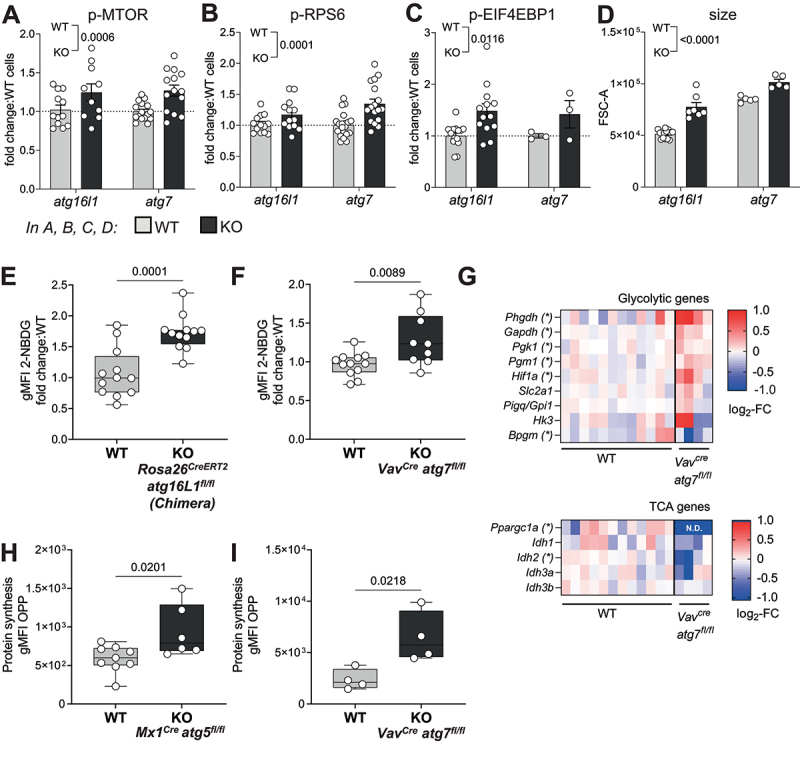
Increased AA uptake in HSCs results in MTOR activation, cell growth, upregulation of glycolysis and protein translation. (**A-C**) MTOR activity measured in HSCs from *Rosa26*^*CreERT2*^ *atg16l*^*fl/fl*^ (as in fig. S2A) and *Vav*^*Cre*^ *atg7*^*fl/fl*^ mice. (**A**) Phosphorylation of MTOR (p-MTOR), (**B**) Phosphorylation of RPS6 (p-RPS6) and (**C**) Phosphorylation of EIF4EBP1 (p-EIF4EBP1) were assessed by flow cytometry. Fold change in gMFI compared with *WT* littermates is shown. *n* = 3–20 mice per group. Two-way ANOVA with post hoc Sidak’s test. (**D**) Relative HSC cell size was measured by forward scatter (FSC) by flow cytometry in *Rosa26*^*CreERT2*^ *atg16l1*^*fl/fl*^ and *Vav*^*Cre*^ *atg7*^*fl/fl*^ mice. Two-way ANOVA with post hoc Sidak’s test. (**E, F**) Glucose uptake was assessed by fluorescent 2-NBDG using flow cytometry in HSCs from *Rosa26*^*CreERT2*^ *atg16l1*^*fl/fl*^ mixed BM chimeras (E, *n* = 12) or *Vav*^*Cre*^ *atg7*^*fl/fl*^ mice (F, n_wt_ = 12, n_KO_ = 9). Data are represented as mean ± SEM with two-tailed unpaired Student’s *t* test. (**G**) Heatmap of TCA or glycolysis related genes measured by Fluidigm in HSCs from *Vav*^*Cre*^ *atg7*^*fl/fl*^ mice. Data represented as log2-fold change relative to *WT*. (**H, I**) Protein synthesis rate of HSCs was measured using the OPP-Click assay with flow cytometry in HSCs from *Mx1*^*Cre*^ *atg5*^*fl/fl*^ (H; (n_WT_ = 9, n_KO_ = 7) or *Vav*^*Cre*^ *atg7*^*fl/fl*^ (I; *n* = 4) mice. gMFI of OPP data are represented as mean ± SEM with two-tailed unpaired Student’s *t* test. *WT*=Cre^−^ and/or fl/^+^ littermates.

Sensing of high cellular AA content, in particular of leucine, arginine and glutamine, is known to activate the MTOR pathway [28]. We found phosphorylation of MTOR, and its downstream targets EIF4EBP1 and RPS6 to be increased in autophagy-deficient HSCs, both a*tg7* KO or *atg16l1* KO (Figure 3A-C). Because MTOR activation signals for cell growth, glycolysis and translation [2], we further investigated whether these parameters were also altered in autophagy-deficient HSCs. Indeed, autophagy-deficient HSCs exhibited a significant increase in cell size compared to autophagy-sufficient HSCs (Figure 3D). Furthermore, uptake of glucose as measured by 2-NBDG staining (a fluorescent analogue of glucose) was elevated in autophagy-deficient HSCs (Figure 3E,F). This was corroborated by the expression profile of metabolic genes, showing increased transcript levels of classical glycolytic enzymes, but not tricarboxylic acid (TCA) cycle genes, in *atg7*-deficient HSCs when compared to *WT* counterparts (Figure 3G). Interestingly, enhanced MTOR activation in autophagy-deficient HSCs was also associated with an increase in protein synthesis measured by the incorporation of labeled puromycin (O-propargyl-puromycin (OPP) click assay) [9] in both *atg5* KO and *atg7* KO mice (Figure 3H,I). We reproduced the same effects on MTOR activation, translation and Kyn uptake in a non-cytopenic context using *Fgd5*^*CreERT2*^ *atg16l1*^*fl/fl*^ mice (Fig. S3A-C).

As loss of autophagy correlates with higher proliferation rates in autophagy-deficient cells [18], we evaluated whether any changes in HSC metabolism could also be caused by fluoracil (5FU)-induced cell cycling in cells from *WT* mice. We observed that 5FU treatment expectedly induced HSC proliferation (Fig. S3E) and that this was accompanied by higher expression of phosphorylated RPS6 (p-RPS6) and increased translation (Fig. S3F,G). Furthermore, HSCs from 5FU-treated animals exhibited higher expression of SLC3A2/CD98 (Fig. S3H), higher uptake of cystine (as an alternative method to measure AA uptake), but unchanged uptake of Kyn (Fig. S3 I,J). As cystine and Kyn are preferentially imported by SLC7A11/xCT and SLC7A5/LAT-1, AA transporters that dimerize with SLC3A2/CD98, this suggests that 5FU-driven proliferation selectively modulates AA uptake. We followed up analyzing whether proliferation is also increased in autophagy-deficient HSCs in a non-cytopenic context, using *Fdg5*^*CreERT2*^ *atg16l1*^*fl/fl*^ mice. Similar to results previously obtained in *Vav*^*Cre*^ *atg7*^*fl/fl*^ mice, *atg16l1* KO (Tomato^+^) were more proliferative than their *WT* counterparts (Tomato^−^, Fig. S3D), suggesting that autophagy-deficiency can induce HSC proliferation in a cell-intrinsic manner and might contribute to the observed metabolic changes. Overall, we detected robust activation of theMTOR pathway when autophagy is lost in HSCs, which led to typical downstream MTOR-mediated metabolic remodeling.

### Rapamycin normalizes the metabolic status of autophagy deficient HSCs

**Figure 4.**
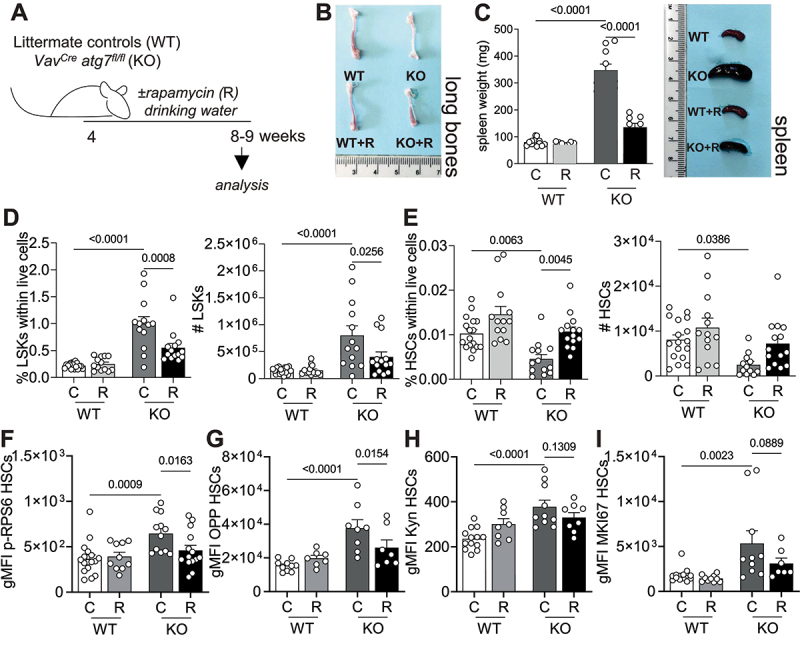
Rapamycin normalizes the metabolic status of autophagy deficient HSCs. (A) Experimental set-up of rapamycin (R) treatment in *Vav*^*Cre*^ *atg7*^*fl/fl*^ mice. (B) Representative images of long bones. (C) Weight and representative images of spleens. (D) LSK frequencies within live cells (left panel) and absolute numbers (right panel). (E) HSC frequencies within live (left panel) cells and absolute numbers (right panel). D-E: (n_WT_ = 17, n_WT+R_ = 13, n_KO_ = 13, n_KO+R_ = 14) (F) gMFI of p-RPS6 within HSCs (n_WT_ = 17, n_WT+R_ = 10, n_KO_ = 13, n_KO+R_ = 14). (G) gMFI of OPP within HSCs as a readout of translation (n_WT_ = 10, n_WT+R_ = 7, n_KO_ = 8, n_KO+R_ = 7). (H) gMFI of Kyn as a readout of AA uptake within HSCs (n_WT_ = 13, n_WT+R_ = 8, n_KO_ = 10, n_KO+R_ = 8). (I) gMFI of MKI67 within HSCs (n_WT_ = 13, n_WT+R_ = 10, n_KO_ = 10, n_KO+R_ = 7). D-E: pooled data from 3–5 independent experiments. Data are represented as mean ± SEM with two-way ANOVA with post hoc Tukey’s test.

As MTOR activation and its downstream effects, in particular increased translation [9], are detrimental to the long-term regeneration potential of HSCs, we sought to determine, by reversing MTOR activation with rapamycin [1], whether this is a maladaptation involved in the autophagy deficiency-induced loss of HSCs. We treated *Vav*^*Cre*^ *atg7*^*fl/fl*^ mice with rapamycin [1] in the drinking water from 4 to 8–9 weeks of age (Figure 4A). Notably, rapamycin treatment alleviated the key symptoms typically observed in *atg7* KO mice [18], such as anemia in long bones and splenomegaly (Figure 4B,C). Moreover, the enhanced frequencies and absolute numbers of LSKs and reduction in HSCs in autophagy-deficient mice were reversed upon rapamycin treatment (Figure 4D,E). As expected, rapamycin prevented excessive mTOR activation, measured by p-RPS6 (Figure 4F) and translation (Figure 4G), in autophagy-deficient HSCs. However, uptake of AA was not significantly affected by rapamycin treatment (Figure 4H), suggesting that the increased uptake of AAs might be upstream of MTOR activation in these cells.

We then addressed whether the beneficial effects of rapamycin could be explained by modulation of proliferation speed by assessing the proliferation profiles of HSCs from *Vav*^*Cre*^ *atg7*^*fl/fl*^ mice treated or not with rapamycin. As observed in Tomato^+^ HSCs from *Fdg5*^*CreERT2*^ *atg16l1*^*fl/fl*^ mice and confirming previous reports, autophagy-deficient cells were more proliferative than their wild type counterparts (Figure 4I). However, rapamycin treatment was not able to revert this phenotype, which suggests that proliferation is not the determining cause of loss of stemness driven by exacerbated anabolism in HSCs (Figure 4I). Because MYC contributes to glycolysis, GLUT-1 expression, cell proliferation and has been shown to be upregulated in *atg7*-deficient T cells [29], we also assessed the expression of *Myc* transcripts in LSKs from *Vav*^*Cre*^ *atg7*^*fl/fl*^ mice treated or not with rapamycin in drinking water. However, we did not observe any significant changes in *Myc* expression caused by autophagy-deficiency or rapamycin treatment in LSKs (Fig. S4A).

Moreover, we wanted to assess the impact of MTOR inhibition by rapamycin on the expression of AA transporters. As ATF4 (activating transcription factor 4) is known to regulate AA transporter expression [30], we assessed the expression of *Atf4* transcripts in LSKs from *Vav*^*Cre*^ *atg7*^*fl/fl*^ mice. Interestingly, *Atf4* expression was higher in autophagy-deficient LSKs, a phenotype that was reversed upon rapamycin treatment (Fig. S4B). To evaluate whether autophagy-deficiency leads to a similar phenotype of SLC7A11/xCT and SLC7A5/LAT-1 selective modulation as observed in 5FU-driven proliferation, we determined the transcriptional expression of *Slc3a2*, *Slc7a11* and *Slc7a5* in LSKs sorted from *Vav*^*Cre*^ *atg7*^*fl/fl*^ mice (Fig. S4C). We took *Slc1a5* as a positive control of autophagy regulation, as SLC1A5/ASCT2 was the AA transporter mostly upregulated in *atg7*-deficient cells in a similar experimental setup (Figure 2C). Autophagy-deficient cells showed an increased expression of all tested AA transporters. Intriguingly, MTOR inhibition by rapamycin promoted strong downregulation of transcripts for these transporters, opposed to its impact on AA uptake (Fig. S4C). These results suggest that MTOR contributes to the transcriptional regulation of expression of AA transporters in LSKs, but that this does not reflect on functional changes in AA uptake in autophagy-deficient cells.

**Figure 5.**
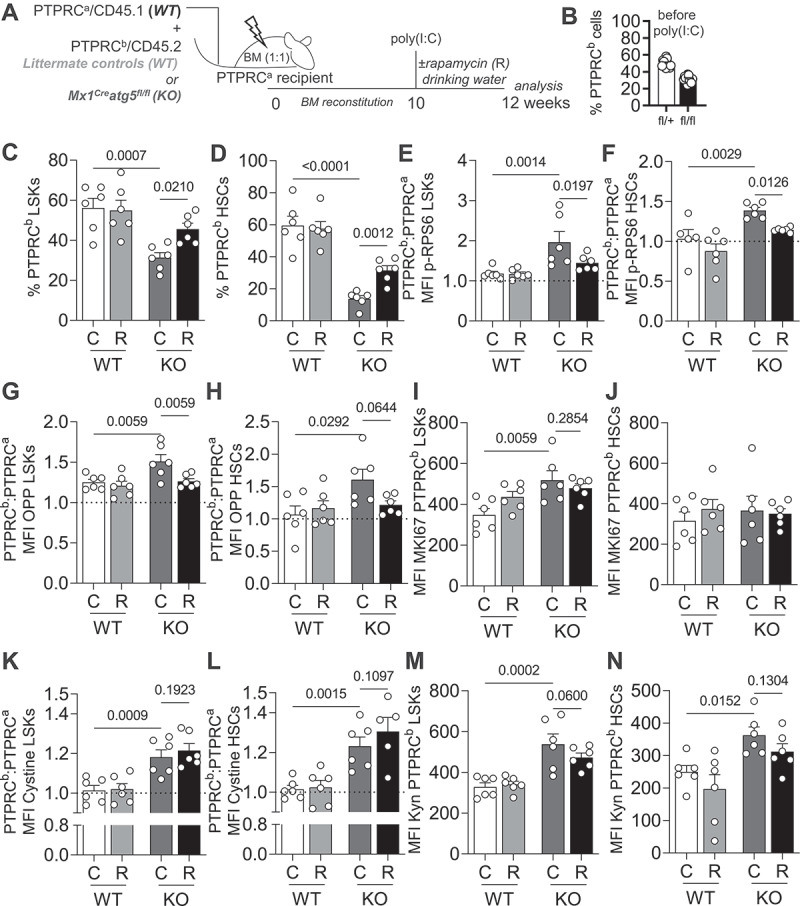
The positive effect of MTOR inhibition on autophagy deficient HSCs is cell intrinsic. (A) Experimental setup of rapamycin treatment in *Mx1*^*Cre*^ *atg5*^*fl/fl*^:*WT* (1:1) mixed BM chimeras. (B) Frequencies of PTPRC^b^/CD45.2 cells in the blood of chimeric mice 10 weeks after reconstitution, prior to poly(I:C) treatment. (C) Frequencies of PTPRC^b^/CD45.2 LSKs. (D) Frequencies of PTPRC^b^/CD45.2 HSCs. (E) MFI of p-RPS6 (PTPRC^b^/CD45.2: PTPRC^a^/CD45.1 ratio) in LSKs. (F) MFI of p-RPS6 (PTPRC^b^/CD45.2: PTPRC^a^/CD45.1 ratio) in HSCs. (G) MFI of OPP (PTPRC^b^/CD45.2: PTPRC^a^/CD45.1 ratio) in LSKs. (H) MFI of OPP (PTPRC^b^/CD45.2: PTPRC^a^/CD45.1 ratio) in HSCs. (I) MFI of MKI67 in PTPRC^b^/CD45.2 LSKs. (J) MFI of MKI67 in PTPRC^b^/CD45.2 HSCs. (K) MFI of cystine-FITC (PTPRC^b^/CD45.2: PTPRC^a^/CD45.1 ratio) in LSKs. (L) MFI of cystine-FITC (PTPRC^b^/CD45.2: PTPRC^a^/CD45.1 ratio) in HSCs. (M) MFI of Kyn in PTPRC^b^/CD45.2 LSKs. (N) MFI of Kyn in PTPRC^b^/CD45.2 HSCs. Pooled data from 2 experiments (*n* = 6 animals/group). Data are represented as mean ± SEM with two-way ANOVA with post hoc Tukey’s test.

Finally, to exclude any potential effects of rapamycin on progenitors and/or mature hematopoietic cells, we also treated mixed BM chimera with rapamycin (PTPRC^b^/CD45.2 *Mx1*^*Cre*^ *atg5*^*fl/fl*^ or *Mx1*^*Cre*^ *Atg5*^*fl/+*^ BM mixed with PTPRC^a^/CD45.1 *WT* BM; Figure 5A,B). Analysis of donor-derived PTPRC^b^/CD45.2 *atg5* KO HSCs confirmed that inflation of the LSK compartment is not observed in a non-cytopenic setup and that rapamycin can rescue HSC loss (Figure 5C,D). Expectedly, rapamycin treatment reduced p-RPS6 levels in LSKs and HSCs (Figure 5E,F) and also fully reversed translation to *WT* levels (Figure 5G,H). As observed in *Vav*^*Cre*^ *atg7*^*fl/fl*^ mice, rapamycin treatment had no impact on proliferation (Figure 5I,J) or AA uptake (Figure 5K-N). We also performed the same experiment treating mice with rapamycin intraperitoneally (Fig. S5) and had comparable results. Corroborating our observations using a second non-cytopenic setup, rapamycin treatment also rescued the loss of HSCs in *Fgd5*^*CreERT2*^ *atg16L1*^*fl/fl*^ mice (Fig. S6), highlighting the cell-intrinsic effects of rapamycin on HSC health.

**Figure 6.**
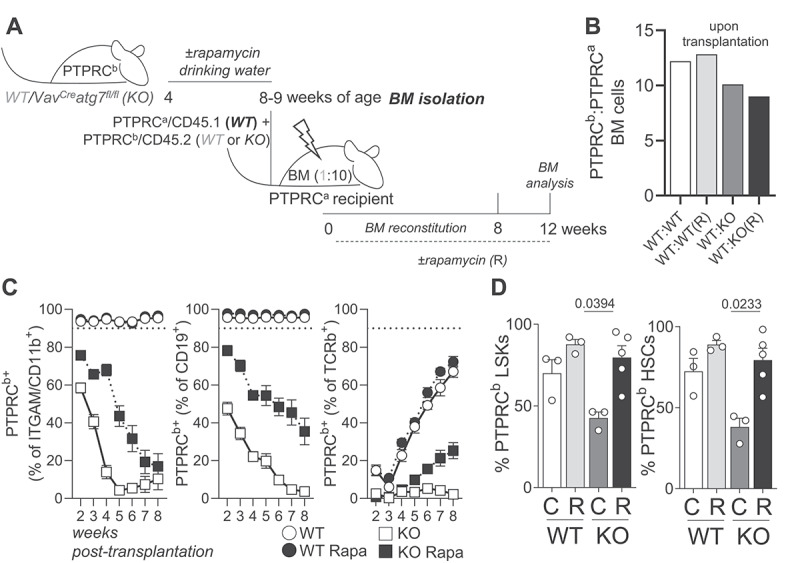
Rapamycin has a positive impact on autophagy-deficient HSC function upon transplantation. (A) Experimental setup. (B) Ratios of PTPRC^b^/CD45.2: PTPRC^a^/CD45.1 WT cells prior to BM transplantation. (C) Contribution of *WT* (PTPRC^a^/CD45.1) and autophagy deficient *Vav*^*Cre*^ *atg7*^*fl/fl*^ or *WT* (PTPRC^b^/CD45.2) cells to the myeloid (ITGAM/CD11b^+^), B cell (CD19^+^) and T cell (TCRb^+^) compartments in recipient mice (*n* = 8–10/animals per group). (D) Frequencies of PTPRC^b^/CD45.2 LSKs and HSCs in the BM of recipient mice 12 weeks after transplantation (*n* = 3–5 animals/group). Data are represented as mean ± SEM with two-tailed unpaired Student’s *t* test.

Transplantation of *Vav*^*Cre*^ *atg7*^*fl/fl*^ HSCs to new hosts leads to complete loss of these cells within weeks [18]. Aiming to evaluate whether rapamycin also improves hematopoietic reconstitution, we isolated BM cells from *Vav*^*Cre*^*atg7*^*fl/fl*^ mice or wild type littermates treated or not with rapamycin in drinking water and transplanted them into new hosts (Figure 6A,B). To ensure the survival of the mice and that any outcomes would be cell-intrinsic, we performed this experiment in a chimeric setting. We found that rapamycin-treatment in autophagy-deficient HSCs led to an improved peripheral hematopoietic reconstitution (Figure 6C). Moreover, rapamycin-treated autophagy-deficient HSCs remained present at comparable frequencies to *WT* HSCs in the bone marrow (Figure 6D). These results suggest that rapamycin treatment improves metabolic health, stemness and multilineage reconstitution capacity of autophagy-deficient HSCs. Thus, we propose that the enhanced MTOR activation observed in autophagy-deficient HSCs is maladaptive and secondary to the loss of autophagy, thereby contributing to the exhaustion of autophagy-deficient HSCs.

## Discussion

Previous studies of mice with autophagy-deficient hematopoiesis [18,19] could not establish to what degree the observed HSC loss and deficiencies were due to HSC-intrinsic or -extrinsic mechanisms. Here, using mice with bone marrow chimeric for normal and autophagy-deficient hematopoiesis, as well as through HSC-specific deletion of critical autophagy components, we are the first to definitely demonstrate that HSC homeostasis depends on HSC-intrinsic autophagy. Moreover, this study provides novel mechanistic insights into the intrinsic role of autophagy in HSCs. Until now, most studies pursued the hypothesis that autophagy’s role is to degrade unwanted material in stem cells, in particular mitochondria. While this makes sense as stem cells are typically quiescent cells, which therefore cannot dilute defunct material to daughter cells [18,19,31], we here provide experimental evidence that autophagy also has an important role in regulating provision of macromolecules to stem cells. We showed that upon loss of autophagy, HSCs increased the expression of AA transporters on the cell surface, resulting in an excessive influx of AA into the cell. This was followed by MTOR activation, leading to increased cell size, glucose uptake, protein synthesis and, eventually, HSC loss. Autophagy-deficient HSCs could be rescued with rapamycin indicating that the observed increased MTOR activity in autophagy-deficient HSCs is a maladaptation to autophagy loss.

We observed increased expression of different amino acid transporters in autophagy-deficient LSKs (using different Atg deletions and Cre systems): SLC1A5/ASCT2, SLC38A1/SNAT1, SLC38A2/SNAT2, SLC7A11/xCT and SLC7A5/LAT-1. These amino acid transporters are antiporters and symporters of neutral and/or anionic amino acids across the plasma membrane. In a*tg16L1* KO cells, we also observed increased expression of SLC26A6/PAT1, which localizes to the lysosomes. Taken together this suggests that autophagy loss has a broad and nonselective impact on AA transporter expression, resulting in higher AA uptake (measured by both Kyn and cystine uptake). We reason that higher MTOR activation observed in autophagy-deficient HSCs, when compared to autophagy-competent cells, is a consequence of increased AA transporter expression and AA uptake. This is in line with several studies showing that influx of leucine, arginine or glutamine directly stimulates MTORC1 activity [32–34]. Conversely and in support of this, in *slc1a5*^*-/-*^*/asct2*^−/−^ leukemic cells, MTORC1 is inhibited as evidenced by decreased levels of p-RPS6 and 2-NBDG uptake [35]. Similarly, in human hepatoma cells, SLC1A5/ASCT2 silencing inhibits MTORC1, which in turn signals to the translational machinery [36]. Together with our study, where we observed that MTOR inhibition leads to decreased expressions of transcripts for AA transporters, this establishes a firm link between AA transporter expression and MTOR function. Our results also provide further evidence of AA transporter expression and uptake being upstream of MTOR activation, as autophagy-deficient cells can have MTOR activation inhibited by rapamycin treatment but exhibit unchanged levels of Kyn and cystine uptake.

Our data suggests that autophagy depletion leads to cell surface AA transporter upregulation and several mechanisms can contribute to this phenotype. Firstly, the increased glucose uptake could also be a MTOR-independent phenomenon, but rather a direct consequence of mitophagy loss to compensate for diminished ATP generation by mitochondrial respiration, as found in several hematopoietic cells [37–39]. This would subsequently result in increased levels of intracellular lactate, which can stimulate SLC1A5/ASCT2 expression via HIF1A (hypoxia inducible factor 1 subunit alpha) and MYC/c-MYC [40]. However, we did not observe significant changes in *Myc* expression (Fig. S4A). Secondly, a possible signaling mechanism was revealed in a cancer cell line in which AA transporter gene expression and AA uptake increased in autophagy-deficient cancer cells under conditions of glutamine deprivation [30]. It involved the interaction between SIRT6 (sirtuin 6) and ATF4, a transcription factor that controls AA transporter expression. We observed higher expression of *Atf4* transcripts in autophagy-deficient LSKs, a phenotype that was reversed upon rapamycin treatment (Fig. S4B). This suggests that MTORC1 may regulate transcriptional levels of AA transporters also via ATF4. In contrast to our findings in HSCs, however, in cancer lines the AA transporter upregulation is beneficial and feeding glutamine to the cells rescues their viability [30]. We predict that these contrasting results might be explained by the fact that highly proliferative cancer cells thrive on activated MTORC1 whereas HSCs do not. Thirdly and most importantly based on our results, autophagy might play a role in AA transporter degradation. Indeed, an autophagic route for SLC38A2/SNAT2 degradation has recently been described [41]. We found that MTOR inhibition by rapamycin treatment has an impact on the transcriptional regulation of AA transporters, but that this does not reflect in less AA uptake (measured by both Kyn and cystine import). These discrepancies between transcriptional and functional modulation of AA uptake support the hypothesis that autophagy plays a role in AA transporters’ degradation. Although MTOR inhibition resulted in lower transcripts for AA transporters and *Atf4*, a positive regulator of their expression, there was still a continuous influx of amino acids. In untreated autophagy-deficient cells, this culminated in exacerbated MTOR activation and translation. While upon rapamycin treatment the influx of AA was not changed, the detrimental effects downstream of MTOR on HSC stemness were neutralized, resulting in better HSC function.

In many tissues, such as white adipose tissue or muscle, the phenotypes of mice with hyperactive MTORC1 share several features with those of defective autophagy. These phenotypes can be largely rescued with rapamycin [28]. This has been explained by the fact that inhibiting MTORC1 with rapamycin induces the early steps of autophagy. Similarly, a recent report revealed that MTOR-mediated regulation of HSC cell size is detrimental to HSC fitness and reconstitution potential, which can similarly be rescued by rapamycin treatment [42]. Here we show that rapamycin reversed these phenotypes even in autophagy-deficient HSCs. Previous work suggested that the bidirectional transport of AAs, especially L-glutamine efflux in exchange for leucine, regulates MTOR, translation and autophagy [35]. This is in line with our hypothesis that upregulation of AA transporters and AA uptake lead to MTOR activation.

Interestingly, in regulatory T (Treg) cells, another hematopoietic cell type in which autophagy plays a central role, autophagy deficiency also upregulates MTORC1, MYC and glycolysis, which contribute to defective Treg function. Exposure to rapamycin largely restores the expression of the Treg transcription factor FOXP3 in *atg7*-deficient Treg cells [29]. Therefore, autophagy maintains Treg cell stability via limiting MTOR activity.

Evidence is increasing that impaired autophagic flux contributes to pathologies. For example, with age, autophagy levels decrease in a proportion of HSCs [19] and autophagy prevents aging in immune cells [43]. Interestingly, rapamycin improves HSC function in old mice [1]. Patients with a germline mutation of *Atg7* (and diminished autophagy) have recently been described but have not been analyzed for their HSC function and blood lineages [44]. Other patients with mutations in autophagy-related genes (e.g *LRBA*, *TTP2* and *EPG5* in Vici syndrome) show variable immune system abnormalities [45–47]. Our data suggest the tantalizing possibility that low dose rapamycin might improve immune deficiencies and pathological conditions in these patients.

## Materials and methods

### Mouse models

All animal work was reviewed and approved by Oxford Ethical committee and the UK Home office under the project licenses PPL30/3388 and P01275425. *Vav*^*Cre*^ *atg7*^*fl/fl*^ [21], *Mx1*^*Cre*^ *atg5*^*fl/fl*^ [20], *Rosa26*^*ERT2Cre*^ *atg16L1*^*fl/fl*^ Rosa26-stop-tdTomato (new model), *Fgd5*^*CreERT2*^*atg16L1*^*fl/fl*^ Rosa26-stop-tdTomato [26], and B6.SJL/J PTPRC^a^/CD45.1 mice were bred in-house and housed on a 12 h dark:light cycle and mouse were fed *ad libitum*, under specific pathogen-free (SPF) conditions. Littermate controls were either heterozygous for the floxed gene or Cre negative, as no obvious differences were found between these. For all inducible models, controls were also injected with Tamoxifen (Sigma-Aldrich, T5648-1 G) or poly(I:C) (Sigma-Aldrich, P9582-5 MG) respectively. For all experiments and phenotypic analyses, data from male and female mice were pooled as no obvious difference was found between sexes. Tamoxifen-induced deletion of *Atg* genes in *Rosa26*^*ERT2Cre*^ *atg16L1*^*fl/fl*^ and *Fgd5*^*CreERT2*^*atg16L1*^*fl/fl*^ mice was performed from 8–10 weeks of age by oral gavage of tamoxifen (5 mg/mouse/day for 5 consecutive days). *Mx1*^*Cre*^ *atg5*^*fl/fl*^ mice were injected intraperitoneally every other day for 1 week with 250 μg poly(I:C) in 250 μL saline or 250 μL phosphate-buffered saline (PBS; Sigma-Aldrich, D8537) to induce deletion of *Atg5*. Mice were monitored over the period of indicated days or weeks and peripheral blood and BM cells were collected for analysis. Rapamycin (Alpha-Aesar, J62473.MC) treatment of *Mx1*^*Cre*^ *atg5*^*fl/fl*^: PTPRC^a^/CD45.1 *WT* chimeras was intraperitoneally as previously described [48]. *VavCre atg7*^*fl/fl*^ rapamycin treatment was done from 4 weeks of age by diluting it into drinking water (50 ug/ml).

### Bone marrow chimera

Bone marrow (BM) cells were collected by crushing the femur, tibia and hips of the donor mice (PTPRC^a^/CD45.1 or PTPRC^b^/CD45.2). The recipient B6.SJL/J PTPRC^a^/CD45.1 mice were lethally irradiated with a total of 11 Gy in two equal doses 4 h apart. 2 to 24 h after the second irradiation a total of 2 × 10^6^ BM cells (ratio of 1:1 or 1:10 of PTPRC^a^/CD45.1 *WT* and PTPRC^b^/CD45.2 genetically modified or *WT* cells) in a total volume of 200 μL PBS, were injected intravenously into recipient mice. Peripheral blood analyses were conducted post-transplantation to check the BM reconstitution.

### Fluidigm gene expression analysis

HSCs from littermate controls or *Vav*^*Cre*^ *atg7*^*fl/fl*^ were flow-sorted (100 cells) into OneStep lysis buffer. RNA was reverse transcribed and cDNA was pre-amplified using the CellsDirect OneStep q-RT kit (Invitrogen, 10043982). The selected genes were amplified and analyzed for expression using a dynamic 48 × 48array (Biomark Fluidigm, BMK-M-48.48GT) as previously described [49]. Data were analyzed using the 2-DDCt method, and all results were normalized to *Actb*, *B2m* and *Hprt* expression after which the best housekeeping gene was selected for further analysis. Biological replicates represent individual mice in each experiment.

### Translation and amino acid uptake assessment

Protein synthesis rate was measured using the Click-iT Plus OPP Protein Synthesis Assay (Thermo Fisher, C10456) according to manufacturer’s protocol. Geometric mean fluorescence intensity (compared to vehicle) was used as an indicator of the relative translation rate. AA uptake in autophagy-deficient HSCs was measured by the flow cytometry-based kynurenine (Kyn) assay as previously described [27], and cystine uptake was assessed using BioTracker Cystine-FITC Live Cell Dye (Sigma-Aldrich, SCT047).

### RT qPCR

RNA was isolated using the RNeasy Micro kit (Qiagen, 74004) and reverse transcribed using the High Capacity cDNA Reverse Transcription kit (Applied Biosystems, 4368814). qRT-PCR was performed using Taqman Gene Expression Master Mix (Applied Biosystems, 4369016) on a ViiA 7 instrument (Thermo Fisher). Data were analyzed using the 2−ΔCt method, and all results were normalized to *Actb*.

### Flow cytometry

BM cells were washed with ice-cold PBS. Cells were stained with fixable Live/Dead staining (Invitrogen, L34993 or L34957), FcR block (Biolegend, 101302), and surface marker antibodies at 4°C for 20–30 min. Antibodies used were: anti-PTPRC/B220 APC, clone RA36B2 (Biolegend, 103212), anti-ITGAM/CD11b BV785, clone M1/70 (Biolegend, 101243), anti-CD19 APC, clone 1D3 (eBioscience, 25-0193-82), anti-SLAMF1/CD150 BV785, clone TC15-12F12.2 (Biolegend, 115937), anti-CD4 BV605, clone RM4–5 (Biolegend, 100547), anti-PTPRC^a^/CD45.1 FITC, clone A20 (eBioscience, 11-0453-82), anti-PTPRC^a^/CD45.1 PerCPCy5.5, clone A20 (Biolegend, 110728), anti-PTPRC^b^/CD45.2 BV605, clone 104 (Biolegend, 109841), anti-PTPRC^b^/CD45.2 BV711, clone 104 (Biolegend, 109847), anti-BCM1/CD48 AF700, clone HM48–1 (Biolegend, 103426), anti-BCM1/CD48 APC, clone HM48–1 (Biolegend, 103411), α-CD8 AF700, clone 53–6.7 (Biolegend, 100730), anti-SLC3A2/CD98 AF647, clone 4F2 (Biolegend, 128210), anti-KIT/cKIT/CD117 PE-Cy7, clone 2B8 (eBioscience, 25-1171-82), anti-Lineage cocktail (Biolegend, 133303), anti-LY6C BV421, clone HK1.4 (Biolegend, 128031), anti-LY6G PE-Cy7, clone 1A8 (Biolegend, 127618), anti-LY6A/Sca-1 FITC, clone E13–161.7 (Biolegend, 122506), anti-LY6A/Sca-1 PerCP-Cy5.5, clone D7 (Invitrogen, 45-5981-82), anti-LY6A/Sca-1 PB, clone E13–161.7 (Biolegend, 122520), anti-Siglec-F PE, clone S17007L (Biolegend, 155506), anti-SLC1A5/ASCT2 (Cell Signaling Technology, 5345S), anti-SLC26A6/PAT1 (Novus Biologicals, NBP2–93440), anti-SLC38A1/SNAT1 (Novus Biologicals, NBP2–13336), anti-SLC38A2/SNAT2 (Novus Biologicals, NBP1–88872), anti-TCRb PerCPCy5.5, clone H57–597 (eBioscience, 17-5961-82). When staining included unconjugated antibodies, this was followed by incubation with goat anti-rabbit conjugated to PE (Invitrogen, A10542) at 4°C for 20–30 min. Glucose uptake was measured by incubating cells for 30 min measurements, cells were incubated in media containing 50 mg/ml 2-NBDG for 20 min at 37°C after surface antibody staining. When intracellular proteins were also analyzed, cells were fixed with BD™ Phosflow Fix Buffer (BD Biosciences, 558049), permeabilized with Permeabilization Buffer (Invitrogen, 00-8333-56) or BD™ Phosphow Perm/Wash I (BD Biosciences, 557885), followed by antibody staining at room temperature for 1 h or at 4°C overnight. Antibodies used were: anti-MKI67 FITC, clone SOIA15 (Invitrogen, 11-5698-82), anti-phospho-EIF4EBP1 PE, clone V3NTY24 (Invitrogen, 12-9107-42), anti-phospho-MTOR eF660, clone MRRBY (Invitrogen, 50-9718-42), anti-phospho-RPS6 eF450, clone cupk43k (Invitrogen, 48-9007-41), anti-phospho-RPS6 APC, clone cupk43k (Invitrogen, 17-9007-42). After washing with PBS, cells were acquired with four-laser LSR or Fortessa X-20 (BD Biosciences) and analyzed using FlowJo 10.8.0/10.8.1.

### Statistical analysis

To test if data point values were in a Gaussian distribution, a normality test was performed before applying parametric or non-parametric statistical analysis. When two groups were compared, unpaired Student’s *t* test or Mann-Whitney test were applied. When comparisons were done across more than two experimental groups, analysis were performed using Two-Way ANOVA with post hoc Tukey’s test or Sidak multiple testing correction. *P* values were considered significant when < 0.05, and exact *P* values are provided in the figures. All analyses were done using GraphPad Prism 9 software. Identical or similar experiments were performed at least twice. If not indicated, data are represented pooled from at least two experiments.

## Supplementary Material

Supplemental Material

## References

[1] Chen C, Liu Y, Liu Y, et al. mTOR regulation and therapeutic rejuvenation of aging hematopoietic stem cells. Sci Signal. 2009;2:ra75. doi: 10.1126/scisignal.2000559 19934433 PMC4020596

[2] Saxton RA, Sabatini DM. mTOR signaling in growth, metabolism, and disease. Cell. 2017;168(6):960–976. doi: 10.1016/j.cell.2017.02.004 28283069 PMC5394987

[3] Meng D, Frank AR, Jewell JL. mTOR signaling in stem and progenitor cells. Development. 2018;145(1). doi: 10.1242/dev.152595 PMC5825873 29311260

[4] Wilson A, Laurenti E, Oser G, et al. Hematopoietic stem cells reversibly switch from dormancy to self-renewal during homeostasis and repair. Cell. 2008;135(6):1118–1129. doi: 10.1016/j.cell.2008.10.048 19062086

[5] Simsek T, Kocabas F, Zheng J, et al. The distinct metabolic profile of hematopoietic stem cells reflects their location in a hypoxic niche. Cell Stem Cell. 2010;7(3):380–390. doi: 10.1016/j.stem.2010.07.011 20804973 PMC4159713

[6] Takubo K, Nagamatsu G, Kobayashi CI, et al. Regulation of glycolysis by Pdk functions as a metabolic checkpoint for cell cycle quiescence in hematopoietic stem cells. Cell Stem Cell. 2013;12(1):49–61. doi: 10.1016/j.stem.2012.10.011 23290136 PMC6592822

[7] Ito K, Carracedo A, Weiss D, et al. A PML–PPAR-δ pathway for fatty acid oxidation regulates hematopoietic stem cell maintenance. Nature Med. 2012;18(9):1350–1358. doi: 10.1038/nm.2882 22902876 PMC3566224

[8] Vannini N, Girotra M, Naveiras O, et al. Specification of haematopoietic stem cell fate via modulation of mitochondrial activity. Nat Commun. 2016;7(1):13125. doi: 10.1038/ncomms13125 27731316 PMC5064016

[9] Signer RA, Magee JA, Salic A, et al. Haematopoietic stem cells require a highly regulated protein synthesis rate. Nature. 2014;509(7498):49–54. doi: 10.1038/nature13035 24670665 PMC4015626

[10] Buszczak M, Signer RJ, Morrison SJ. Cellular differences in protein synthesis regulate tissue homeostasis. Cell. 2014;159(2):242–251. doi: 10.1016/j.cell.2014.09.016 25303523 PMC4222182

[11] Chua BA, Van Der Werf I, Jamieson C, et al. Post-transcriptional regulation of homeostatic, stressed, and malignant stem cells. Cell Stem Cell. 2020;26:138–159. doi: 10.1016/j.stem.2020.01.005 32032524 PMC7158223

[12] Girotra M, Monnard C, Konz T, et al. Mineral and amino acid profiling of different hematopoietic populations from the mouse bone marrow. Int J Mol Sci. 2020;21(17):6444. doi: 10.3390/ijms21176444 32899421 PMC7504538

[13] Richter FC, Obba S, Simon AK. Local exchange of metabolites shapes immunity. Immunology. 2018;155(3):309–319. doi: 10.1111/imm.12978 29972686 PMC6187213

[14] Taya Y, Ota Y, Wilkinson AC, et al. Depleting dietary valine permits nonmyeloablative mouse hematopoietic stem cell transplantation. Science. 2016;354:1152–1155. doi: 10.1126/science.aag3145 27934766

[15] Oburoglu L, Tardito S, Fritz V, et al. Glucose and glutamine metabolism regulate human hematopoietic stem cell lineage specification. Cell Stem Cell. 2014;15(2):169–184. doi: 10.1016/j.stem.2014.06.002 24953180

[16] Poncet N, Taylor PM. The role of amino acid transporters in nutrition. Curr Opin Clin Nutr Metab Care. 2013;16(1):57–65. doi: 10.1097/MCO.0b013e32835a885c 23196813

[17] van Galen P, Kreso A, Mbong N, et al. The unfolded protein response governs integrity of the haematopoietic stem-cell pool during stress. Nature. 2014;510(7504):268–272. doi: 10.1038/nature13228 24776803

[18] Mortensen M, Soilleux EJ, Djordjevic G, et al. The autophagy protein Atg7 is essential for hematopoietic stem cell maintenance. J Exp Med. 2011;208:455–467. doi: 10.1084/jem.20101145 21339326 PMC3058574

[19] Ho TT, Warr MR, Adelman ER, et al. Autophagy maintains the metabolism and function of young and old stem cells. Nature. 2017;543:205–210. doi: 10.1038/nature21388 28241143 PMC5344718

[20] Warr MR, Binnewies M, Flach J, et al. FOXO3A directs a protective autophagy program in haematopoietic stem cells. Nature. 2013;494:323–327. doi: 10.1038/nature11895 23389440 PMC3579002

[21] Mortensen M, Ferguson DJ, Edelmann M, et al. Loss of autophagy in erythroid cells leads to defective removal of mitochondria and severe anemia in vivo. Proc Natl Acad Sci U S A. 2010;107:832–837. doi: 10.1073/pnas.0913170107 20080761 PMC2818953

[22] Feng W, Chang C, Luo D, et al. Dissection of autophagy in human platelets. Autophagy. 2014;10(4):642–651. doi: 10.4161/auto.27832 24458007 PMC4091151

[23] Pua HH, Dzhagalov I, Chuck M, et al. A critical role for the autophagy gene Atg5 in T cell survival and proliferation. J Exp Med. 2007;204:25–31. doi: 10.1084/jem.20061303 17190837 PMC2118420

[24] Kuhn R, Schwenk F, Aguet M, et al. Inducible gene targeting in mice. Science. 1995;269:1427–1429. doi: 10.1126/science.7660125 7660125

[25] Joseph C, Quach JM, Walkley CR, et al. Deciphering hematopoietic stem cells in their niches: a critical appraisal of genetic models, lineage tracing, and imaging strategies. Cell Stem Cell. 2013;13(5):520–533. doi: 10.1016/j.stem.2013.10.010 24209759

[26] Gazit R, Mandal PK, Ebina W, et al. Fgd5 identifies hematopoietic stem cells in the murine bone marrow. J Exp Med. 2014;211:1315–1331. doi: 10.1084/jem.20130428 24958848 PMC4076584

[27] Sinclair LV, Neyens D, Ramsay G, et al. Single cell analysis of kynurenine and System L amino acid transport in T cells. Nat Commun. 2018;9(1):1981. doi: 10.1038/s41467-018-04366-7 29773791 PMC5958064

[28] Deleyto-Seldas N, Efeyan A. The mTOR-Autophagy axis and the control of metabolism. Front Cell Dev Biol. 2021;9:655731. doi: 10.3389/fcell.2021.655731 34277603 PMC8281972

[29] Wei J, Long L, Yang K, et al. Autophagy enforces functional integrity of regulatory T cells by coupling environmental cues and metabolic homeostasis. Nat Immunol. 2016;17(3):277–285. doi: 10.1038/ni.3365 26808230 PMC4755832

[30] Zhang N, Yang X, Yuan F, et al. Increased amino acid uptake Supports autophagy-deficient cell survival upon glutamine deprivation. Cell Rep. 2018;23:3006–3020. doi: 10.1016/j.celrep.2018.05.006 29874586

[31] Guan JL, Simon AK, Prescott M, et al. Autophagy in stem cells. Autophagy. 2013;9(6):830–849. doi: 10.4161/auto.24132 23486312 PMC3672294

[32] Jewell JL, Kim YC, Russell RC, et al. Metabolism. Differential regulation of mTORC1 by leucine and glutamine. Science. 2015;347:194–198. doi: 10.1126/science.1259472 25567907 PMC4384888

[33] Chantranupong L, Scaria SM, Saxton RA, et al. The CASTOR proteins are arginine sensors for the mTORC1 pathway. Cell. 2016;165(1):153–164. doi: 10.1016/j.cell.2016.02.035 26972053 PMC4808398

[34] Chen J, Ou Y, Luo R, et al. SAR1B senses leucine levels to regulate mTORC1 signalling. Nature. 2021;596(7871):281–284. doi: 10.1038/s41586-021-03768-w 34290409

[35] Nicklin P, Bergman P, Zhang B, et al. Bidirectional transport of amino acids regulates mTOR and autophagy. Cell. 2009;136(3):521–534. doi: 10.1016/j.cell.2008.11.044 19203585 PMC3733119

[36] Fuchs BC, Finger RE, Onan MC, et al. ASCT2 silencing regulates mammalian target-of-rapamycin growth and survival signaling in human hepatoma cells. Am J Physiol Cell Physiol. 2007;293:C55–63. doi: 10.1152/ajpcell.00330.2006 17329400

[37] Stranks AJ, Hansen AL, Panse I, et al. Autophagy controls acquisition of aging features in macrophages. J Innate Immun. 2015;7(4):375–391. doi: 10.1159/000370112 25764971 PMC4386145

[38] Riffelmacher T, Clarke A, Richter FC, et al. Autophagy-dependent generation of Free fatty acids is critical for normal neutrophil differentiation. Immunity. 2017;47(3):466–480 e465. doi: 10.1016/j.immuni.2017.08.005 28916263 PMC5610174

[39] Puleston DJ, Zhang H, Powell TJ, et al. Autophagy is a critical regulator of memory CD8(+) T cell formation. Elife. 2014;3: doi: 10.7554/eLife.03706 PMC4225493 25385531

[40] Perez-Escuredo J, Dadhich RK, Dhup S, et al. Lactate promotes glutamine uptake and metabolism in oxidative cancer cells. Cell Cycle. 2016;15(1):72–83. doi: 10.1080/15384101.2015.1120930 26636483 PMC4825768

[41] Morotti M, Zois CE, El-Ansari R, et al. Increased expression of glutamine transporter SNAT2/SLC38A2 promotes glutamine dependence and oxidative stress resistance, and is associated with worse prognosis in triple-negative breast cancer. Br J Cancer. 2021;124:494–505. doi: 10.1038/s41416-020-01113-y 33028955 PMC7852531

[42] Lengefeld J, Cheng CW, Maretich P, et al. Cell size is a determinant of stem cell potential during aging. Sci Adv. 2021;7:eabk0271. doi: 10.1126/sciadv.abk0271 34767451 PMC8589318

[43] Zhang H, Puleston DJ, Simon AK. Autophagy and immune senescence. Trends Mol Med. 2016;22:671–686. doi: 10.1016/j.molmed.2016.06.001 27395769

[44] Collier JJ, Guissart C, Olahova M, et al. Developmental consequences of defective ATG7-mediated autophagy in humans. N Engl J Med. 2021;384(25):2406–2417. doi: 10.1056/NEJMoa1915722 34161705 PMC7611730

[45] Lu W, Zhang Y, McDonald DO, et al. Dual proteolytic pathways govern glycolysis and immune competence. Cell. 2014;159(7):1578–1590. doi: 10.1016/j.cell.2014.12.001 25525876 PMC4297473

[46] Lopez-Herrera G, Tampella G, Pan-Hammarstrom Q, et al. Deleterious mutations in LRBA are associated with a syndrome of immune deficiency and autoimmunity. Am J Hum Genet. 2012;90:986–1001. doi: 10.1016/j.ajhg.2012.04.015 22608502 PMC3370280

[47] Finocchi A, Angelino G, Cantarutti N, et al. Immunodeficiency in Vici syndrome: a heterogeneous phenotype. Am J Med Genet A. 2012;158A:434–439. doi: 10.1002/ajmg.a.34244 21965116

[48] Bitto A, Ito TK, Pineda VV, et al. Transient rapamycin treatment can increase lifespan and healthspan in middle-aged mice. Elife. 2016;5. doi: 10.7554/eLife.16351. PMC4996648 27549339

[49] Tehranchi R, Woll PS, Anderson K, et al. Persistent malignant stem cells in del(5q) myelodysplasia in remission. N Engl J Med. 2010;363(11):1025–1037. doi: 10.1056/NEJMoa0912228 20825315

