# Supplementary material for: Autophagy preserves hematopoietic stem cells by restraining MTORC1-mediated cellular anabolism
Source: Autophagy. 2023 Aug 23;20(1):45–57. doi: 10.1080/15548627.2023.2247310 (PMC10761185; doi:10.1080/15548627.2023.2247310)
Supplement: Supplemental Material [file KAUP_A_2247310_SM1626.docx]

# **Supplementary Materials and Methods**

*Western blot*

Following (Z)-4-hydroxytamoxifen (Sigma-Aldrich, H7904-5MG) treatment for 24 h and/or bafilomycin A_1_ (BafA) treatment (10 nM) for 2 h or not, cells were washed with PBS and lysed in RIPA lysis buffer (Sigma-Aldrich, R0278) supplemented with complete Protease Inhibitor Cocktail (Roche, 11697498001) and PhosSTOP (Roche, 4906837001). Protein concentration was calculated by using the BCA Assay (Thermo Scientific, 23225). Samples were diluted in 4x Laemmli Sample Buffer (Bio-Rad, 1610747) and boiled at 100 °C for 5 min. Twenty  µg protein per sample were used for SDS-PAGE analysis. NuPAGE Novex 4%–12% Bis-Tris gradient gel (Invitrogen, NP0335BOX) with MOPS running buffer (Invitrogen, NP0001) was used. Proteins were transferred to a PVDF membrane (Merck Millipore, IPFL00010) and blocked with 5% skimmed milk-TBST (TBS 10x [Sigma-Aldrich, T5912]) diluted to 1x in distilled water containing 0.1% Tween 20 [Sigma-Aldrich, P1379]) for 1h. Membranes were incubated at 4°C overnight with primary antibodies diluted in 1% skimmed milk-TBST and at room temperature for 1 h with secondary antibodies diluted in 1% skimmed milk-TBST supplemented 0.01% SDS. Primary antibodies used were: anti-ATG16L1, clone EPR15638 (Abcam, ab187671) and anti-GAPDH, clone 6C5 (Sigma-Aldrich, MAB374). Secondary antibodies used were: IRDye 680LT Goat anti-Mouse IgG (H + L) (Licor, 926-680-70) and IRDye 800CW Goat anti-Rabbit IgG (H + L) (Licor, 926-322-11). Images were acquired using the Odyssey CLx Imaging System. Data were analyzed using Image Studio Lite or Fiji.

*Autophagy flux*

Autophagy flux was measured using the Guava® Autophagy LC3 Antibody-based Detection Kit (Luminex, FCCH100171). As the only modification to manufacturer’s instructions, autophagy inhibition was achieved by BafA (10 nM) treatment for 2 h.

*5FU treatment*

5FU (fluoracil) (LKT laboratories, F4480) induced proliferation was performed by intravenous administration (3 mg) in C57BL/6 mice with 4 weeks of age.

**Supplementary Figures**

**
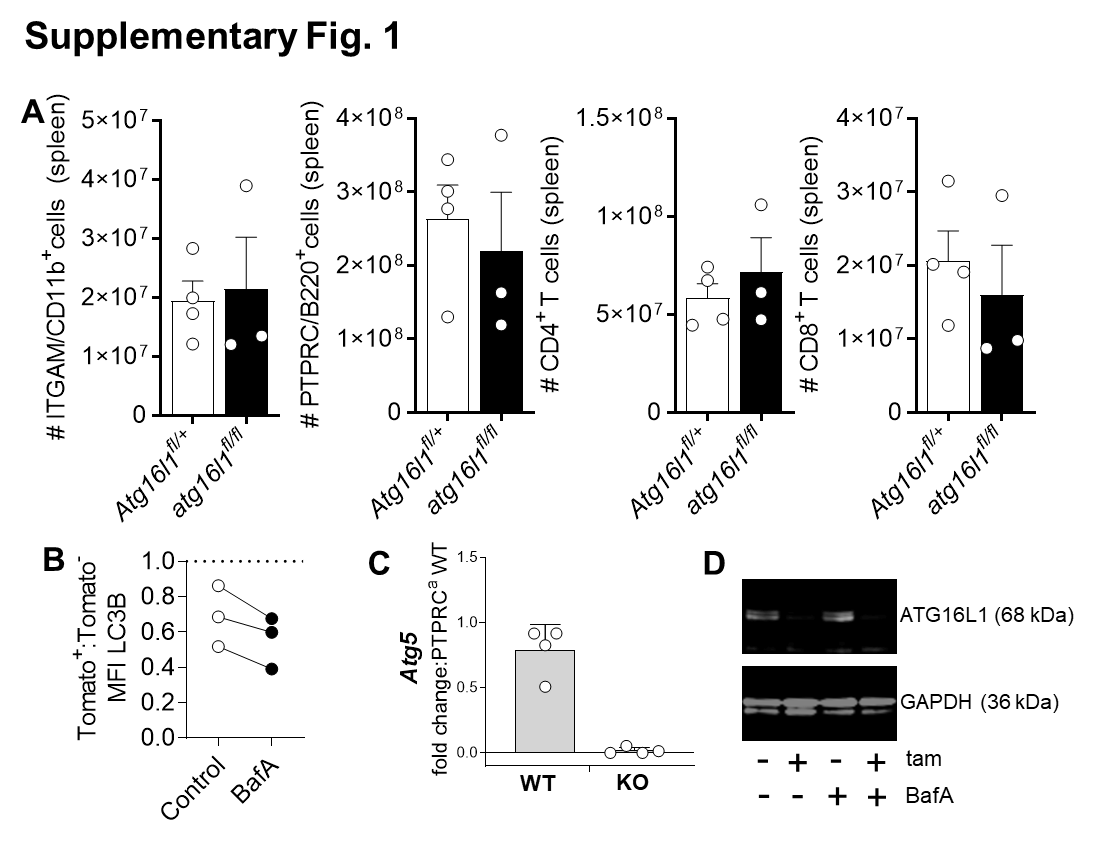
**

**Figure S1.** *Atg16l1* deletion does not lead to cytopenia and inhibits autophagy flux in the Fgd5 model and tamoxifen-driven deletion of *Atg5* and *Atg16l1* is efficient. (**A**) *Fgd5^CreERT2^ atg16l1^fl/fl^* mice were analyzed 5 weeks after tamoxifen treatment (n_WT_=4, n_KO_=3). Splenic frequencies of myeloid cells (ITGAM/CD11b^+^), PTPRC/B220^+^ cells, CD4^+^ T cells and CD8^+^ T cells are depicted. (**B**) Ratio (Tomato^+^:Tomato^-^) of LC3B expression in HSCs from *Fgd5^CreERT2^ atg16l1^fl/fl^* mice exposed or not to BafA treatment (10 nM for 2 h) (n=3). (**C**) Real-time qPCR analysis of *Atg5* gene expression in sorted LSKs from *Mx1^Cre^ atg5^fl/fl^* chimera; fold change to PTPRC^a^/CD45.1 WT cells. Data are shown as fold change normalized to *Actb* (n=4). (**D**) ATG16L1 protein expression in sorted T cells from *Rosa26^CreERT2^ atg16L1^fl/f^*^l^ mice 24 h following tamoxifen treatment *in vitro* assessed by immunoblotting (tam=tamoxifen; BafA=bafilomycin A_1_). Data representative of 1 out of 4 experiments.

##

## **
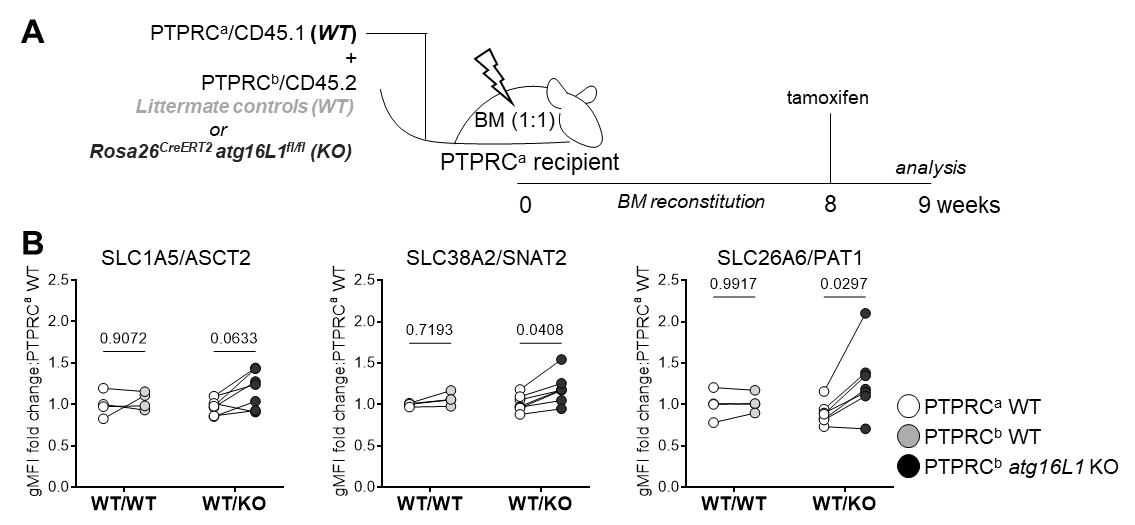
**

## **Figure S2.** Expression of amino acid transporters is upregulated in constitutive model of autophagy deficiency. (**A**) Experimental setup for generation of mixed BM chimeras using the inducible *Rosa26^CreERT2^ atg16l1^fl/fl^* model. (**B**) Surface expression of AAT (amino acid transporters) in HSCs from mixed *Rosa26^CreERT2^ atg16l1^fl/^*^fl^:*WT* (1:1) BM chimera were analyzed by flow cytometry. PTPRC^a^/CD45.1 WT (white symbols), PTPRC^b^/CD45.2 WT (gray symbols), and PTPRC^b^/CD45.2 *atg16L1* KO (black symbols). Geometric mean of fluorescence intensity (gMFI) of AAT is normalized and expressed as fold change to WT PTPRC^a^/CD45.1. n_WT:WT_ =7 and n_WT:KO_=12 per group with Two-way ANOVA with Sidak’s correction for multiple testing.

## **
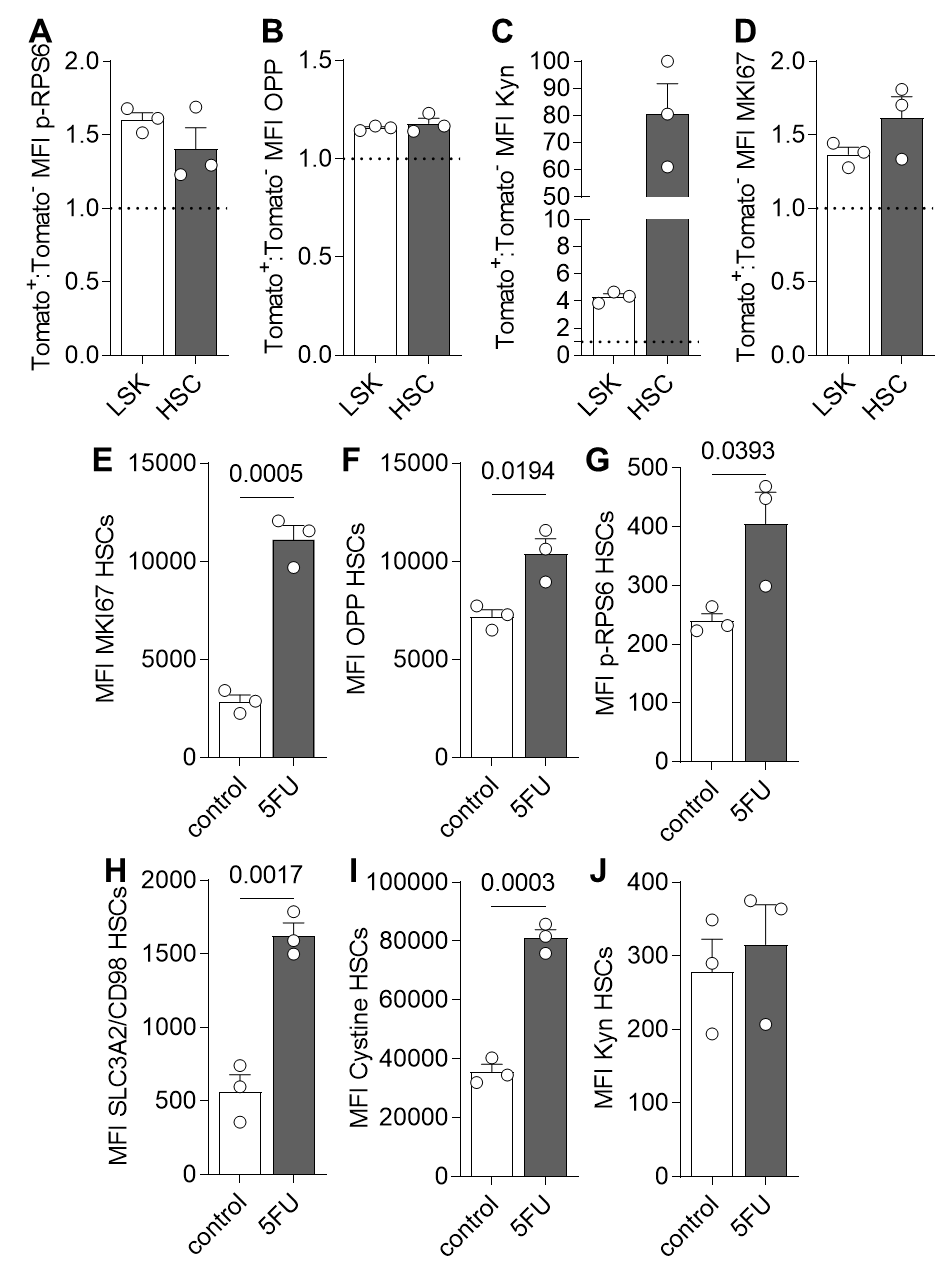
**

## **Figure S3.** Increased AA uptake in autophagy deficient HSCs from *Fgd5^CreERT2^ atg16l1^fl/fl^* mice results in MTOR activation, translation and proliferation. In Tomato^+^ *atg16l1* KO HSCs (**A**) Fold change of p-RPS6 expression, as a readout of MTOR activation, (**B**) Fold change of OPP expression, as a readout for translation, (**C**) Fold change of kynurenine (Kyn) expression, as a readout for amino acid uptake, **(D)** Fold change of MKI67 expression, as a readout for proliferation. Dotted line is *WT* (Tomato^-^) levels in cells from 3 mice (average). Data was obtained from cells isolated from *Fgd5^CreERT2^ atg16L1^fl/fl^* mice 5 weeks post-tamoxifen treatment (n=3). 5FU-induced proliferation increased AA uptake, MTOR activation and translation in HSCs from wild type animals. Mice received 3mg of 5FU intravenously and BM was isolated 7 days later (n_control_=3; n_5FU_=3). Proliferation, measured by MKI67 (**E**), translation, measured by OPP (**F**), MTOR activation, measured by p-RPS6 (**G**), SLC3A2/CD98 expression (**H**) and amino acid uptake (**I, J**) were assessed by flow cytometry.


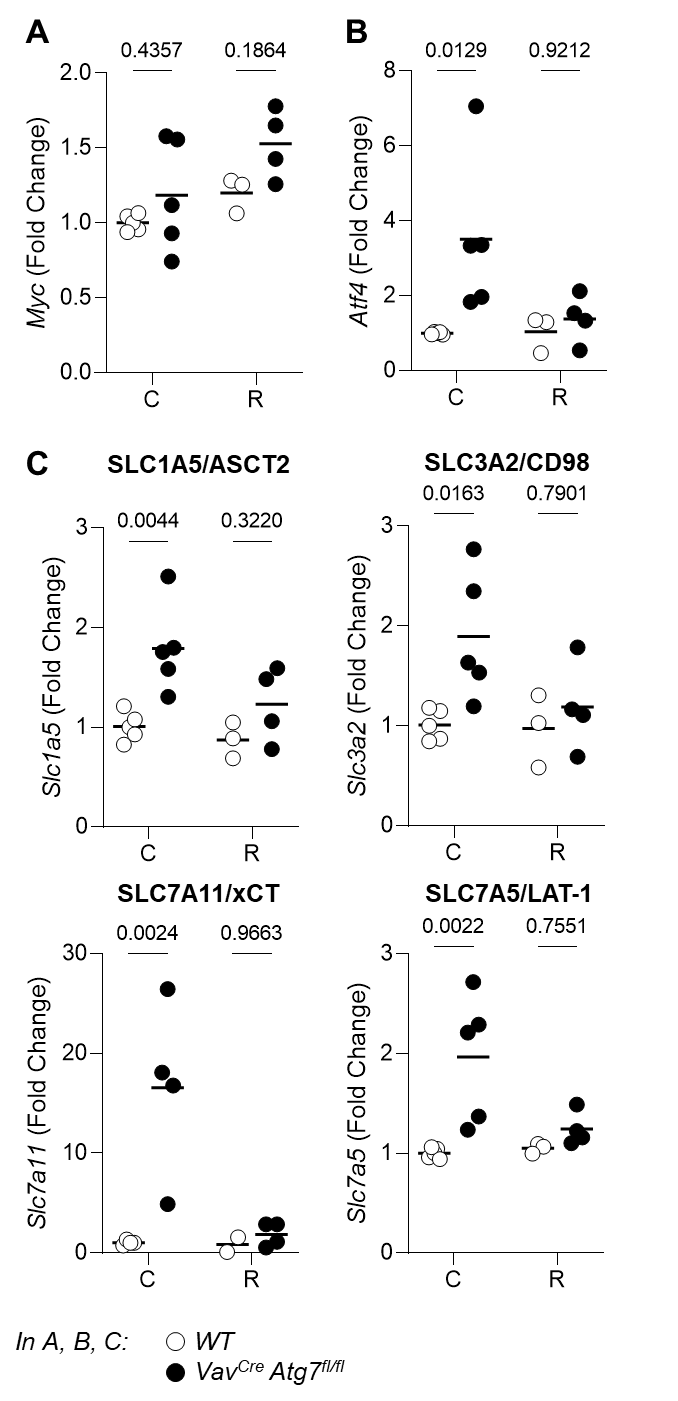


**Figure S4.** mRNA expression of metabolic regulators and amino acid transporters in HSCs from *Vav^Cre^ atg7^fl/fl^* mice. Real-time qPCR analysis of *Myc* (**A**), *Atf4* (**B**), and *Slc1a5* (encoding for SLC1A5/ASCT2), *Slc3a2*, *Slc7a11* and *Slc7a5* (AA transporters, **C**) expression in sorted LSKs from *Vav^Cre^ atg7^fl/fl^* mice. SLC3A2/CD98, SLC7A11/xCT and SLC7A5/LAT-1 are involved in cystine and kynurenine uptake. R= rapamycin treated animals. Data are relative to *Actb* (n=2-5/group). Two-way ANOVA with post hoc Sidak’s test.


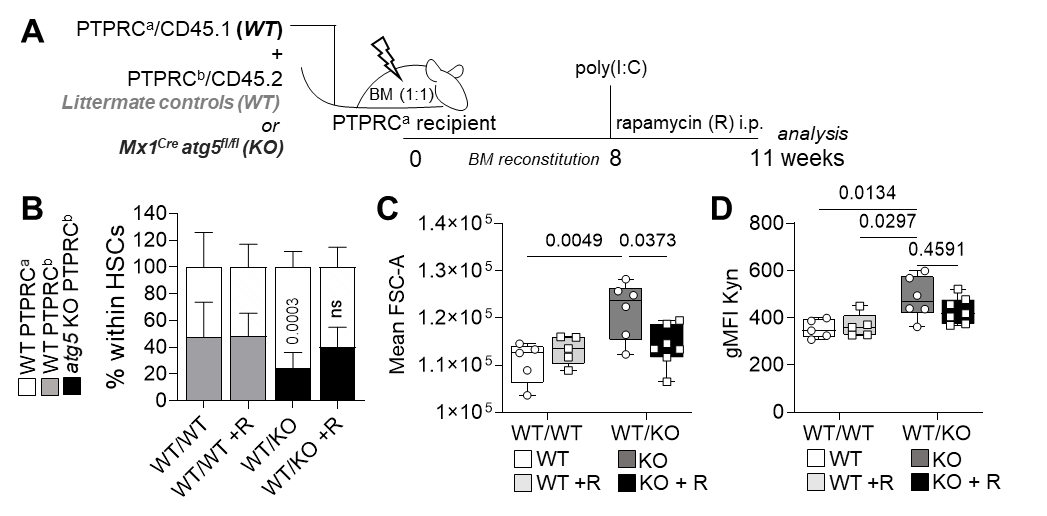


**Figure S5.** The rescue effect of MTOR inhibition on autophagy deficient HSCs is cell intrinsic and the phenotype of higher AA transporter protein expression is not reversed upon rapamycin treatment. (**A**) Experimental setup of rapamycin (R) treatment in *Mx1^Cre^ atg5^fl/fl^*:WT (1:1) mixed BM chimeras. (**B**) Contribution of donor-derived PTPRC^b^/CD45.2 HSCs (WT in gray, KO in black) and recipient-derived PTPRC^a^/CD45.1 WT HSC (in white) to the total HSC compartment. Multiple unpaired Student’s *t* test used. (**C**) HSC cell size measured as mean FSC intensity. (**D**) gMFI of kynurenine (Kyn) uptake in HSCs. n=4-7 animals/group. When not otherwise stated, data are represented as mean ± SEM with two-way ANOVA with post hoc Tukey’s test.

## **
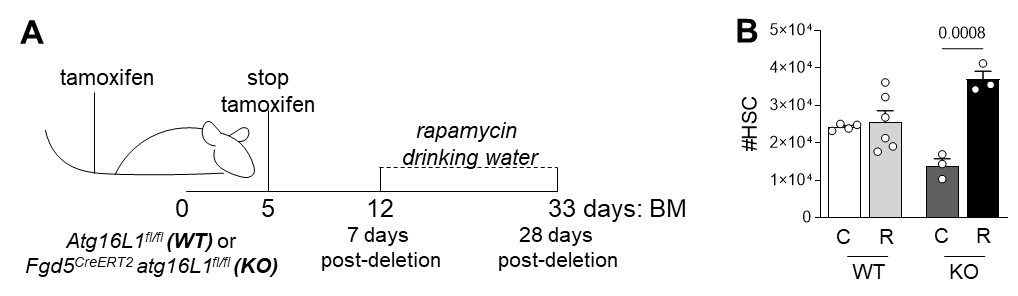
**

## **Figure S6.** Rapamycin rescues HSCs in an inducible HSC-specific mouse model of autophagy deletion. (**A**) Experimental setup for the treatment with rapamycin in mice where *Atg16l1* deletion in HSC is induced by tamoxifen treatment in *Fdg5^CreERT2^ atg16^fl/fl^* mice. (**B**) Absolute number of HSCs after treatment with rapamycin (R) (n_WT_=4, n_WT+R_=6, n_KO_=3, n_KO+R_=3). Representative data from one experiment.
